# Frequent allelic deletion at the FHIT locus associated with p53 overexpression in squamous cell carcinoma subtype of Taiwanese non-small-cell lung cancers

**DOI:** 10.1038/sj.bjc.6601778

**Published:** 2004-05-04

**Authors:** Y-C Lee, C-T Wu, J-Y Shih, Y-S Jou, Y-L Chang

**Affiliations:** 1 Department of Surgery, National Taiwan University Hospital and National Taiwan University College of Medicine, Taipei 100, Taiwan; 2 Department of Pathology, National Taiwan University Hospital and National Taiwan University College of Medicine, Taipei 100, Taiwan; 3 Department of Internal Medicine, National Taiwan University Hospital and National Taiwan University College of Medicine, Taipei 100, Taiwan; 4 Division of Molecular and Genomic Medicine, National Health Research Institutes, Taipei, Taiwan

**Keywords:** FHIT, loss of heterozygosity, lung cancers

## Abstract

The fragile histidine triad (FHIT) gene, encompassing the FRA3B fragile site at chromosome 3p14.2, is a tumour suppressor gene involved in different tumour types including non-small-cell lung cancers (NSCLCs). In the current study, we examined for allelic deletion at the FHIT locus in 58 primary and microdissected NSCLCs, for which a clinicopathologic profile was available. We found a loss of 87.7% in heterozygosity (LOH) frequency at one or more microsatellite markers (D3S1289, D3S2408, D3S1766, D3S1312, D3S1600). Allelic deletion of D3S1766 was related to tumour histology in 10 of 11 squamous cell carcinomas (90.9%) displaying LOH compared with nine of 17 adenocarcinomas (52.9%; *P*=0.049). Besides, in the subset of adenocarcinomas, a higher rate of LOH at D3S1289 was observed in male (six out of eight, 75%) than in female patients (four out of 17, 23.5%; *P*=0.028). However, FHIT LOH was not correlated overall with a variety of clinical parameters including sex, smoking status, staging, lymph node metastasis and survival. These results indicated that the high frequency of FHIT gene disruption was important in the development of both squamous cell carcinomas and adenocarcinomas. Furthermore, there was no association between LOH at FHIT and protein expression, suggesting the presence of complex mechanisms of Fhit inactivation. On the other hand, the association between FHIT LOH and p53 protein overexpression assessment reached statistical significance (*P*=0.026), implying that common alterations affect the two genes in tumour progression.

Lung cancer is the most common cause of cancer death in Taiwan (Lee *et al*, 2002) and in Western countries (Geradts *et al*, 2000). With only around 10% of 5-year survival rate, lung cancer caused more than 6000 deaths per year in Taiwan. The majority of these tumours are non-small-cell carcinomas (NSCLCs), of which adenocarcinoma is the most common histological type (Chen *et al*, 1990; Ihde and Minna, 1991). Cigarette smoking is the major risk factor. However, other environmental and genetic factors have been reported to be associated with the increasing risk of lung cancer, including HPV 16 of 18 virus infection, water and air pollution, cooking fume exposure and polymorphisms of cancer susceptible genes.

Field cancerisation and multistep tumorigenesis are two concepts that are essential in understanding the underlying mechanisms of carcinogenesis of the aerodigestive tract. Field cancerisation was first proposed by Slaughter and Skejkal (1953), who suggested that a whole tissue field exposed to common carcinogens, such as cigarette smoke, is at risk for the development of malignancy due to diffuse injury over time. Multistep tumorigenesis refers to the multistep process in which genetic events accumulate and result in malignant transformation (Kinzler and Vogelstein, 1996).

Recent advances in the molecular genetics of human cancers have revealed that multiple tumour suppressor genes are involved in lung carcinogenesis (Yokota and Sugimura, 1993). After the cloning of the fragile histidine triad (FHIT) gene at 3p14.2 (Ohta *et al*, 1996), subsequent genetics studies demonstrated frequent allelic deletion and aberrant FHIT transcripts were observed in primary lung cancers (Sozzi *et al*, 1996; Fong *et al*, 1997) and cell lines of small-cell and non-small-cell types (Yanagisawa *et al*, 1996). The evidence that FHIT suppresses tumorigenicity in cancer cells (Siprashvili *et al*, 1997) supports the contention that FHIT is a tumour suppressor gene. This, together with the recent observation (Sozzi *et al*, 1997) that there is more FHIT allelic loss in carcinomas from smokers than from nonsmokers, strengthens the case for its involvement in the multistage development of lung cancer.

There are several reports on the correlation between abnormalities of the FHIT gene and clinicopathologic features in lung cancers. Loss of heterozygosity (LOH) at the FHIT gene locus in adenocarcinoma was less frequent than that in squamous cell carcinoma (Burke *et al*, 1998). The correlation between LOH at the FHIT gene locus and the patients' survival was different in different studies (Fong *et al*, 1997; Sozzi *et al*, 1997, 1998; Burke *et al*, 1998; Tomizawa *et al*, 1998). Loss of heterozygosity at the FHIT gene locus in smokers has occurred more frequently than that in nonsmokers (Sozzi *et al*, 1997; Zienolddiny *et al*, 2001). It was also reported that lack of Fhit staining correlates with LOH at the FHIT 3p14.2 locus, but not at other loci on 3p (Geradts *et al*, 2000).

The wild-type p53 protein acts as a transcriptional factor in turning on the expression of a DNA damage programme by blocking cell cycle progression in the late G1 phase and by triggering apoptosis in response to stress signals, including DNA damage, hypoxia and nucleotide deprivation (Prives and Hall, 1999). Abnormalities of p53 expression by immunostaining were frequently found in lung cancer including 40–70% of SCLCs and 40–60% of NSCLCs. Previous reports, in our study of Taiwanese NSCLCs, have also indicated that p53 nuclear expression by immunostaining analysis detected more frequent stainable expression in squamous cell carcinoma than that in adenocarcinoma (Chang *et al*, 2003) In addition, p53 overexpression and the loss of Fhit expression has been significantly more common in tumours from smokers than those of nonsmokers (Chang *et al*, 2003).

Since frequent LOH is a hallmark of genomic instability and commonly used as a genetic marker for cancer, we aimed to conduct microsatellite analysis for allelic deletion of FHIT locus for examining the genetic mechanism of allelic deletion with loss of Fhit protein expression. The results of allelic deletion were further characterised for correlation with the clinicopathologic features, including sex, smoking history, histologic types and differentiation, stage, lymph node metastasis and survival. Furthermore, immunohistochemical detection of Fhit and p53 proteins was also compared.

## MATERIALS AND METHODS

### Lung cancer patients and specimens

In all, 58 lung cancer specimens were obtained from patients who underwent surgical resection for NSCLC at National Taiwan University Hospital. These patients were not treated with neoadjuvant chemotherapy and irradiation therapy. All the specimens were formalin fixed and sectioned for microscopic examination after applying haematoxylin–eosin stain. Histological diagnosis and pathological features were obtained including tumour cell type, direct invasion to surrounding structures and regional lymph node metastasis. Pathological staging was performed according to the international staging system for lung cancer (Mountain, 2002), which is based on tumour size, location and involvement, and the presence of lymph node metastases.

This study included 23 male and 35 female patients, and the mean age was 65 years (ranging from 42 to 80 years). The clinical data of these patients, including sex, age, smoking status, location of the tumour and ensuing distinct metastases after surgery were recorded and correlated with the result of Fhit and p53 proteins expression in each tumour. The four stage IIIB cases included three multifocal adenocarcinomas within one pulmonary lobe, one squamous cell carcinoma with pulmonary artery and aortic invasions. Both of the stage IV cases were multifocal adenocarcinomas involving two pulmonary lobes.

### Allelotyping by microsatellite markers

Microsatellite analysis was performed in those cases for which immunohistochemical analysis had been performed. Sections of the tumour and the adjacent nontumour parts used for immunohistochemistry were deparaffinised and stained with haematoxylin–eosin. Tumour cells and adjacent nontumourous alveolar cells were microdissected from each section. Approximately 500–1000 cells were dissected from each section. After microdissection of the tumour tissue, genomic DNA was isolated from the tumour and normal tissue by proteinase K and extraction using standard protocols (Burke *et al*, 1998). Paired tumour and normal DNA were subjected to polymerase chain reaction (PCR) analysis. A total of five microsatellite polymorphic markers, including three dinucleotide (D3S1289, D3S1312 and D3S1600), one trinucleotide (D3S2408) and one tetranucleotide (D3S1766) markers that span a total region of about 9 Megabase pairs (Mb) of FHIT locus on chromosomes 3, were used for allelotyping. The order of markers and FHIT gene are D3S1289–D3S2408–D3S1766–FHIT–D3S1312–D3S1600 with physical interval of 1.03, 3.06, 0.66, 1.07, 0.72 Mb, respectively, based on the NCBI released human genome sequence build 34. The average interval of these markers was approximately 1.8 Mb. The microsatellite markers were purchased from PE Applied Biosystems (Foster City, CA, USA) and from the set of Multi-Coloured Fluorescent Human MapPairs Markers (Version 8) of Research Genetics (Huntsville, AL, USA). These polymorphic microsatellite markers were labelled with one of the four different fluorescent dyes (FAM, HEX and TET, NED) on one PCR primer to allow a simultaneous analysis of many markers based on the differences in fragment sizes and fluorescent labels. This technique provided a powerful tool with which to interpret allelic loss on a quantitative basis efficiently. Polymerase chain reactions were conducted in a volume of 10 *μ*l using 0.03 *μ*M of fluorescent-labelled 250 *μ*M of each dNTPs, 2 mM MgCl_2_, and 0.5 U of FastStart *Taq* DNA Polymerase (Roche Molecular System, CA, USA) with protocols provided by the manufacturers. Polymerase chain reaction products with different fluorescent labels and fragment sizes were pooled and mixed with internal fluorescent-labelled (TAMRA) molecular weight markers for subsequent electrophoresis using a 3700 automated fluorescent DNA sequencer (PE Applied Biosystems, Foster City, CA, USA). Allele sizing was determined using the software of GeneScan Analysis version 3.7 (ABI PRISM) and Genotyper version 3.7 (ABI PRISM). We used GENOTYPE™ ROX 50-5000 DNA Ladder as the fragment calling standards. For each gel image, we employed two persons for allele sizing of markers independently. For each marker, the allelic loss ratio was calculated by the formula of (*T*1/*T*2)/(*N*1/*N*2), in which *T*1 and *T*2 were the values of the two peaks derived from the tumour and *N*1 and *N*2 are from normal tissues. In the current study, we utilised a more stringent definition for the occurrence of LOH. First, peak values of both allele areas and allele heights were used to calculate the allelic loss ration, and thus two sets of data were generated for each marker. Second, only when both sets of data were >2- or <0.5-fold, instead of the 1.5-fold standard commonly used by other studies, the marker region was considered as an LOH locus (Ko *et al*, 2002).

### Immunohistochemistry

For immunohistochemical demonstration of the Fhit protein expression in the tumour tissue, 4-*μ*m-thick sections from each formalin-fixed, paraffin-embedded tissue block were dewaxed with xylene and rehydrated through a graded series of ethanol.

The sections for IHC of the Fhit protein expression were autoclaved in 0.01 M phosphate citrate buffer (pH 6.9) at 121°C for 3 min and were treated with 3% H_2_O_2_–methanol solution to reduce endogenous peroxidase activity. These were then incubated with normal goat serum to reduce nonspecific antibody binding and were subsequently subjected to the primary antibody reaction. The antibody for Fhit protein (1 : 20) (IBL,Gunma, Japan) was left to react with the sections overnight at 4°C. Detection of the immunoreactive staining was carried out by the avidin–biotin–peroxidase complex method according to the manufacturer's instructions (Dako Corporation, Carpinteria, CA, USA). To check for nonspecific staining by the avidin–biotin–peroxidase complex detection system, the primary antibody was replaced with BSA. The sections were then subjected to a colour reaction with 0.05% 3,3-diaminobenzidine in 0.05 M trishydrochloride (pH 7.6) containing 0.01% H_2_O_2_ and were lightly counterstained with haematoxylin.

For IHC of the p53 protein expression in the tumour tissue, the paraffin-embedded tissue block was treated with 0.3% H_2_O_2_ in methanol to block endogenous peroxidase, and heated in a microwave oven for 20 min for antigen retrieval. The tissue sections were then incubated with normal non-immune goat serum. After blotting the excessive goat serum, the slides were incubated with a specific mouse anti-p53 protein antibody ‘p53 (Ab-6), pantropic’ (diluted 1 : 50) (Oncogene Science, Cambridge, MA, USA) for 1 h at room temperature. After washing with phosphate-buffered solution (PBS) three times, the sections were incubated with bionylated goat anti-mouse antibody for 20 min at room temperature. The sections were again washed three times with PBS, and were then incubated with peroxidase-conjugated streptavidin for 15 min at room temperature. After a third triple washing with PBS, the sections were then stained with 0.05% (3′,3) diaminobenzidine tetrachloride freshly prepared in 0.05 M Tris-Hcl (pH 7.6) containing 0.01% H_2_O_2_. Finally, the sections were counterstained with haematoxylin and then mounted.

Immunostaining was classified in the following two groups according to both intensity and extent: (1) negative, no staining was present or positive staining was detected in <10% of the cells and (2) positive immunostaining was present in ⩾10% of the cells. Two independent pathologists (Y-LC and C-TW) were involved in the assessment of expression.

### Statistical analysis

The correlation between various clinical or pathological parameters and the expression of the FHIT gene was analysed using the Fisher's exact and log-rank tests. All of the statistical tests were two-sided.

## RESULTS

### LOH at the FHIT locus

**Figure 1:**
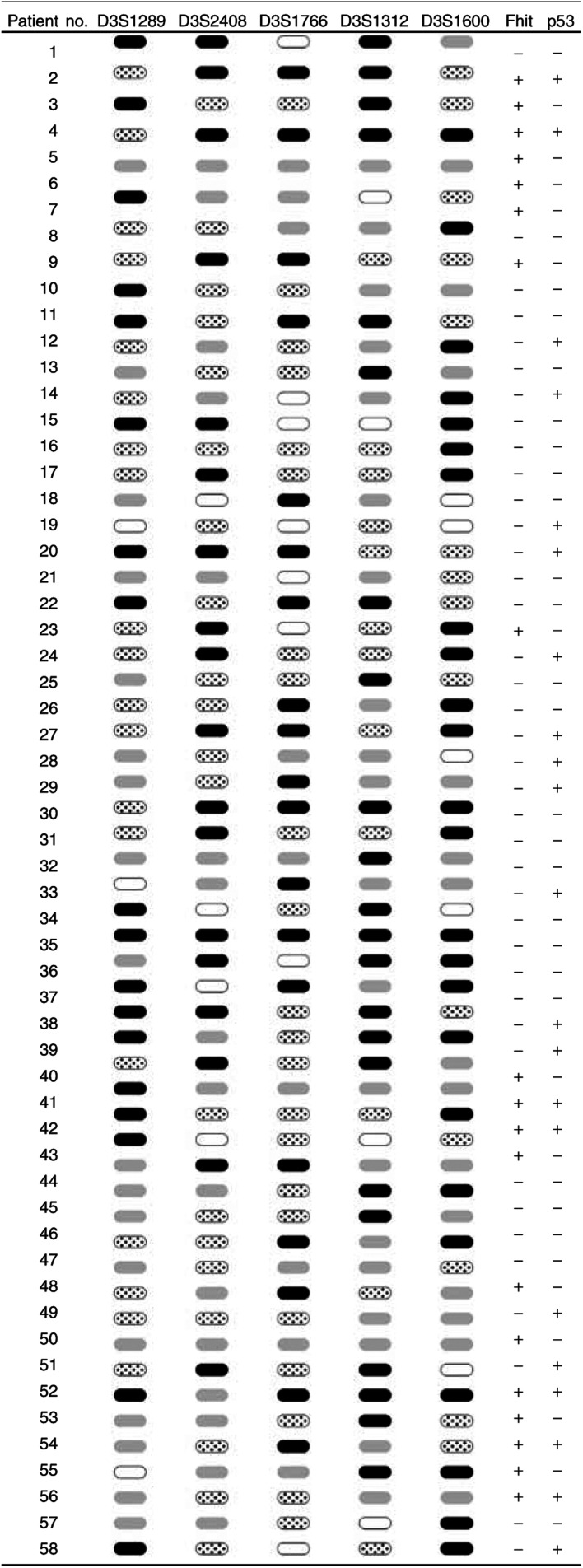
Allelic loss on chromosome 3p14.2 and Fhit and p53 protein expression in 58 cases of Taiwanese NSCLCs. When analysed by five microsatellite markers (i.e. D3S1289, D3S2408, D3S1766, D3S1312 and D3S1600), 50 tumours showed evidence of LOH at one or more sites. 
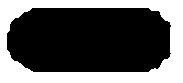
, LOH; 
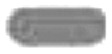
, no LOH; 
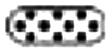
, not informative; 
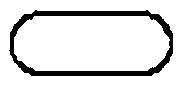
, not analysed. Fhit and p53 (+): ⩾10%, Fhit and p53 (−): <10%.

LOH at the FHIT locus or near the FHIT locus has been found to be strictly associated with abnormal FHIT transcripts in lung tumours (Sozzi *et al*, 1996), therefore loss of one FHIT allele has been considered a crucial step leading to the loss of function of the gene. Tumours and matched normal lung tissues were studied using five microsatellite markers covering a total of 9 Mb and the entire FHIT locus. The normal tissues of all samples were heterozygous for at least one of these markers. Allelic deletion was detected in 50 of 57 (87.7%) informative cases at one or more loci examined when complete data from all five microsatellites were included (Figure 1

).

### Correlations of FHIT alterations with clinicopathologic features and survival

In this cohort of 58 NSCLCs, there was no statistically significant correlation between LOH at the FHIT locus and clinicopathologic features of sex, age, smoking history, stage of disease and lymph node involvement. Likewise, there was no difference in overall survival between patients with LOH-positive and -negative tumours at one or more loci (*P*=0.208).

LOH at D3S1766 was observed more frequently (10 out of 11; 90.9%) in squamous cell carcinomas than in adenocarcinomas (nine out of 17; 52.9%; *P*=0.049) (Table 1

**Table 1:** Relationships between LOH of FHIT and microsatellite makers and clinicopathologic parameters of patient analysed

		**LOH**		
**Makers**	**Clinicopathologic parameters**	**No.of cases with LOH(%)**	**No.of cases without LOH (%)**	***P*-value**
D3S1766	Squamous cell carcinoma	10 (90.9)	1 (9.1)	0.049
	Adenocarcinoma	9 (52.9)	8 (47.1)	
D3S1766	p53 (+)^a^	9 (100)	0 (0)	0.026
	p53 (−)^a^	10 (52.6)	9 (47.4)	
D3S1766	Fhit(+)^a^	6 (54.5)	5 (45.5)	0.409
	Fhit(−)^a^	13 (76.5)	4 (23.5)	
D3S1289	Male in adenocarcinoma	6 (75)	2 (25)	0.028
	Female in adenocarcinoma	4 (23.5)	13 (76.5)	
LOH loci ⩾1	Smoker	20 (83.3)	4 (16.7)	0.439
	Nonsmoker	30 (90.9)	3 (9.1)	

a Immunohistochemical stain, (+): >10%, (−): <10%; LOH= Loss of heterozygosity; FHIT= fragile histidine triad.

). In addition, allelic loss affecting D3S1289 was present in six out of eight (75%) of the adenocarcinomas from male patients, whereas only four out of 17 (23.5%) of the tumours in the female group showed LOH at D3S1289. This difference was statistically significant (*P*=0.028) (Table 1).

### Correlations of LOH at the FHIT locus with Fhit protein expression

Out of 17, 13 (76.5%) tumours with negative Fhit expression demonstrated LOH at the FHIT locus, whereas six out of 11 (54.5%) tumours with positive Fhit expression demonstrated LOH at D3S1766 (Table 1). Thus, there was no statistically significant correlation between LOH at D3S1766 and Fhit expression (*P*=0.409).

### Correlations of LOH at the FHIT locus with p53 protein overexpression

Among 19 cases that showed LOH of FHIT detected by D3S1766, a synergistic effect of p53 overexpression and LOH at FHIT locus was observed in all nine cases (100%) in comparision to none in cases of p53 overexpression and no LOH detected by D3S1766 (*P*=0.026) (Table 1). The nine cases with synergistic effect of LOH at FHIT locus and p53 overexpression were not specifically associated with any subtype of histology (six squamous cell carcinomas and three adenocarcinomas) or smoking history (44%). See supplementary data in Table 1

## DISCUSSION

The FHIT gene and its protein product have been the focus of recent debate with regard to their potential role in tumorigenesis (Croce *et al*, 1999). The FHIT gene is fragile in several tumour types, and FHIT knockout mice models support the idea of a haploinsufficient mechanism for FHIT-promoting tumour growth (Zanesi *et al*, 2001). Therefore, analysis of microsatellite markers is useful in screening for abnormalities of FHIT in tumour cells. In the current study, we performed high-density screening of LOH loci using microsatellite markers to dissect and refine the common LOH loci located on FHIT gene in NSCLC tissues.

Allelic imbalance at the FHIT and the near FHIT loci was assessed with the microsatellite markers D3S1289, D3S2408, D3S1766, D3S1312 and D3S1600. Analysis revealed losses of heterozygosity in at least one FHIT locus in 86.2% of the cases, which was more frequent than the results (22–70%) of previous reports (Sozzi *et al*, 1996; Fong *et al*, 1997; Garinis *et al*, 2001; An *et al*, 2002; Petursdottir *et al*, 2002; Pylkkanen *et al*, 2002). The significantly higher incidence of LOH in NSCLCs from our cases suggest that different somatic or inherited genetic mechanisms could underlie cancer development in these patients. In addition, purified genomic DNA isolated from microdissected DNAs could potentially reduce the masking effects of nontumourous genomic DNA contaminated in tissue specimens and increased LOH frequency. Previous studies have shown that the loss of 3p14.2 is more common in squamous cell carcinomas than in adenocarcinomas (Tsuchiya *et al*, 1992; Testa *et al*, 1994; Zienolddiny *et al*, 2001). Our data are consistent with this association (Table 1).

Interestingly, in the subset of 36 informative adenocarcinomas, a higher LOH rate at D3S1289 was observed in male patients (six out of eight, 75%) than in female patients (four out of 17, 23.5%; *P*=0.028). The high frequency of deletions found at the FHIT locus may indicate a possible role for this locus in male patients with adenocarcinomas.

It has been proposed that the FHIT gene is specifically targeted by carcinogens in cigarette smoke, and a number of reports described an increased frequency of FHIT abnormalities at the DNA or protein level in smokers (Sozzi *et al*, 1997, 1998; Marchetti *et al*, 1998; Nelson *et al*, 1998; Tomizawa *et al*, 1998). Our series of 58 NSCLC patients included 24 smokers and 34 nonsmokers. The frequency of LOH at one or more loci was rather similar among smokers (20 out of 24, 83.3%) and nonsmokers (30 out of 33, 90.9%; *P*=0.439). The lack of correlation between LOH at one or more loci and smoking history are divergent to that of previous results, however, there is no clear explanation for the difference (Burke *et al*, 1998).

In agreement with previous studies (Burke *et al*, 1998; Marchetti *et al*, 1998; Tomizawa *et al*, 1998; Geradts *et al*, 2000), allelic deletion of the FHIT locus did not correlate with sex, age, staging, lymph node metastasis and survival. Thus, our results are consistent with the role of FHIT abnormalities at a relatively early stage of pulmonary neoplasia, rather than in the metastatic progression of invasive lung cancers.

In this study, we did not find a concordance between LOH at the FHIT locus and loss of Fhit expression in NSCLCs. The inconsistency between LOH of FHIT locus and Fhit expression could simply be due to either small deletion or deletion of flanking marker without affecting the transcriptional regulation of FHIT gene. Whereas LOH is generally associated with decreased protein expression, protein inactivation may be due to complex mechanisms other than deletion within an allele (Tanaka *et al*, 1998). Alternatively, abnormal protein expression may occur in the absence of genetic alterations through altered splicing fidelity (Gayther *et al*, 1997). Besides, methylation of FHIT is another important mechanism for loss of Fhit expression (Zöchbauer-Müller *et al*, 2001).

The reported link of FHIT with apoptotic regulation (Ji *et al*, 1999; Sard *et al*, 1999) led us to examine its connection to the status of the p53 protein, a well-characterised antiproliferative factor, which induces growth arrest and apoptosis in response to various stimuli (Prives and Hall, 1999). It was previously indicated that the genetic changes at the FHIT locus may be correlated with p53 mutations (Burke *et al*, 1998; Marchetti *et al*, 1998; Chang *et al*, 2003), while other investigators found no statistically significant correlation between FHIT and p53 abnormalities in NSCLCs (Nelson *et al*, 1998; Geradts *et al*, 2000). By examining the FHIT/p53 profiles, we noticed that the nine cases with synergistic effect of LOH at FHIT locus and p53 overexpression included six squamous cell carcinomas and three adenocarcinomas. And 44% of them were smokers. Since frequent LOH at FHIT locus was detected in smokers of squamous cell carcinoma with p53 aberrations in Western populations (Garinis *et al*, 2001), we speculated that exposure of different carcinogenic agents such as HPV, cooking fume, etc. in Taiwan (Chen *et al*, 1990) might have caused genome instability detected by LOH of FHIT locus in lung cancer patients of different geographic regions. As described by Garinis, the main determinant of tumour growth in FHIT LOH tumours is the state of p53, a finding that strengthens the concept of p53 protecting the cell from the deleterious effects of tumour suppressor gene inactivation or oncogene activation (Garinis *et al*, 2001). Furthermore, the data presented recently constitutes a further molecular evidence of a field cancerisation effect and suggest that specific mutations in p53 and FHIT alteration may occur as an early event of bronchial precancerous lesions (Sozzi *et al*, 2002).

In conclusion, this study showed for the first time that FHIT gene alteration is a very frequent event in both squamous cell carcinomas and adenocarcinomas of the lung despite whatever the smoking status. This unexpectedly high incidence may indicate that specific mechanisms are involved, resulting in frequent deletions in these loci. It may play a role in carcinogenesis of the human lung, and may provide some new approaches to early gene detection for lung cancer. FHIT LOH was not correlated with important clinical parameters, suggesting that it plays a role early in the pathogenesis of lung cancer rather than in later metastatic stages. The particularly frequent FHIT LOH in our NSCLCs and the absence of correlation with Fhit protein expression implies that the presence of complex mechanisms of Fhit inactivation. On the other hand, the significant association between LOH at FHIT and p53 protein overexpression provides evidence of alterations affecting the two genes. As NSCLC is an aggressive disease and shows an unfavourable prognosis, a new treatment such as FHIT gene therapy could represent a desirable breakthrough.

## References

[bib1] An Q, Liu Y, Gao Y, Huang J, Fong X, Liu L, Zhang D, Zhang J, Cheng S (2002) Deletion of tumour suppressor genes in Chinese non-small cell lung cancer. Cancer Lett 184: 189–195 12127691 10.1016/s0304-3835(02)00204-5

[bib2] Burke L, Khan MA, Freedman AN, Gemna A, Rusin M, Guinee DG, Bennett WP, Caporaso NE, Fleming MV, Travis WD, Colby TV, Trastek V, Pairolero PC, Tazelaar HD, Midthun DE, Liotta LA, Harris CC (1998) Allelic deletion analysis of the FHIT gene predicts poor survival in non-small cell lung cancer. Cancer Res 58: 2533–2536 9635574

[bib3] Chang YL, Wu CT, Shih JY, Lee YC (2003) Roles of Fhit and p53 in Taiwanese surgically treated non-small-cell lung cancers. Br J Cancer 89: 320–326 12865924 10.1038/sj.bjc.6601041 PMC2394260

[bib4] Chen CJ, Wu HY, Chuang YC, Chang AS, Luh KT, Chao HH, Chen KY, Chen SG, Lai GM, Huang HH (1990) Epidemiologic characteristics and multiple risk factors of lung cancer in Taiwan. Anticancer Res 10: 971–976 2382996

[bib5] Croce CM, Sozzi G, Huebner K (1999) Role of FHIT in human cancer. J Clin Oncol 17: 1618–1624 10334551 10.1200/JCO.1999.17.5.1618

[bib7] Fong KM, Biesterveld EJ, Virmani A, Wistuba I, Sekido Y, Bader SA, Ahmadian M, Ong ST, Rassoal FV, Zimmerman PV, Giaccone G, Gazdar AF, Minna JD (1997) FHIT and FRA3B 3p14.2 allele loss are common in lung cancer and preneoplastic bronchial lesions and are associated with cancer-related FHIT CDNA splicing aberrations. Cancer Res 57: 2256–2267 9187130

[bib8] Garinis GAa, Gorgoulis VG, Mariatos G, Zacharatos P, Kotsinas A, Liloglou T, Foukas P, Kanavaros P, Kastrinakis NG, Vassilakopoulos T, Vogiatzi T, Field JK, Kittas C (2001) Association of allelic loss at the FHIT locus and p53 alterations with tumour kinetics and chromosomal instability in non-small cell lung carcinomas (NSCLCs). J Pathol 193: 55–65 11169516 10.1002/1096-9896(2000)9999:9999<::AID-PATH731>3.0.CO;2-#

[bib9] Gayther SA, Barski P, Batley SJ, Li L, de Foy KA, Cohen SN, Ponder BA, Caldas C (1997) Aberrant splicing of the TSG 101 and FHIT genes occurs frequently in multiple malignancies and in normal tissues and mimics alterations previously described in tumours. Oncogene 15: 2119–2126 9366528 10.1038/sj.onc.1201591

[bib10] Geradts J, Fong KM, Zimmerman PV, Minna JD (2000) Loss of Fhit expression in non-small-cell lung cancer: correlation with molecular genetic abnormalities and clinicopathological features. Br J Cancer 82: 1191–1197 10735505 10.1054/bjoc.1999.1062 PMC2363352

[bib11] Ihde DC, Minna JD (1991) Non-small cell lung cancer. Part I: biology, diagnosis, and staging. Curr Probl Cancer 15: 65–147 10.1016/0147-0272(91)90014-2 1649734

[bib12] Ji L, Fang B, Yen N, Fong K, Minna JD, Roth JA (1999) Induction of apoptosis and inhibition of tumorigenicity and tumour growth by adenovirus vector-mediated fragile histidine triad (FHIT) gene overexpression. Cancer Res 59: 3333–3339 10416589

[bib13] Kinzler KW, Vogelstein B (1996) Lessons from hereditary colorectal cancer. Cell 87: 159–171 8861899 10.1016/s0092-8674(00)81333-1

[bib14] Ko JY, Lee TC, Hsiao CF, Lin GL, Yen SH, Chen KY, Hsiung CA, Chen PJ, Hsu MM, Jou YS (2002) Definition of three minimal deleted regions by comprehensive allelotyping and mutational screening of FHIT, p16^INK4a^, and 19^ARF^ genes in nasopharyngeal carcinoma. Cancer 94: 1987–1996 11932901 10.1002/cncr.10406

[bib15] Lee YC, Wu CT, Chen JS, Hsu HH, Chang YL (2002) The significance of E-cadherin and *α*-, *β*-, and *γ*-catenin expression in surgically treated non-small cell lung cancers of 3 cm or less in size. J Thorac Cardiovasc Surg 123: 502–506 11882822 10.1067/mtc.2002.119334

[bib16] Marchetti A, Pellegrini S, Bertacca G, Buttitta F, Gaeta P, Carnicelli V, Nardini V, Griseri P, Chella A, Angeletti CA, Bevilacqua G (1998) FHIT and p53 gene abnormalities in bronchioloalveolar carcinomas. Correlations with clinicopathological data and K-ras mutations. J Pathol 184: 240–246 9614374 10.1002/(SICI)1096-9896(199803)184:3<240::AID-PATH20>3.0.CO;2-B

[bib17] Mountain CF (2002) Staging classification of lung cancer. A critical evaluation. Clin Chest Medic 23(1): 103–121 10.1016/s0272-5231(03)00063-7 11901906

[bib18] Nelson HH, Wiencke JK, Gunn L, Wain JC, Christiani DC, Kelsey KT (1998) Chromosome 3p14 alterations in lung cancer: evidence that FHIT exon deletion is a target of tobacco carcinogens and asbestos. Cancer Res 58: 1804–1807 9581816

[bib19] Ohta M, Inoue H, Cotticelli MG, Kastury K, Baffa R, Palazzo J, Siprashvili Z, Mori M, McCue P, Druck T, Croce CM, Huebner K (1996) The FHIT gene, spanning the chromosome 3p14.2 fragile site and renal carcinoma-associated t(3;8) breakpoint, is abnormal in digestive tract cancers. Cell 84: 587–597 8598045 10.1016/s0092-8674(00)81034-x

[bib20] Petursdottir TE, Hafsteinsdottir SH, Jonasson JG, Moller PH, Thorsteinsdottir U, Huiping C, Egilsson V, Ingvarsson S (2002) Loss of heterozygosity at the FHIT gene in different solid human tumours and its association with survival in colorectal cancer patients. Anticancer Res 22: 3205–3212 12530066

[bib21] Prives C, Hall PA (1999) The p53 pathway. J Pathol 187: 112–126 10341712 10.1002/(SICI)1096-9896(199901)187:1<112::AID-PATH250>3.0.CO;2-3

[bib22] Pylkkanen L, Wolff H, Stjernvall T, Tuominen P, Sioris T, Karjalainen A, Anttila S, Husgafvel-Pursiainen K (2002) Reduced Fhit protein expression and loss of heterozygosity at FHIT gene in tumours from smoking and asbestos-exposed lung cancer patients. Int J Oncol 20: 285–290 11788890

[bib23] Sard L, Accornero P, Tornielli S, Delia D, Bunone G, Campiglio M, Colombo MP, Gramegna M, Croce CM, Pierotti M, Sozzi (1999) The tumor-suppressor gene FHIT is involved in the regulation of apoptosis and in cell cycle control. Proc Natl Acad Sci USA 96: 8489–8492 10411902 10.1073/pnas.96.15.8489 PMC17543

[bib24] Siprashvili Z, Sozzi G, Barnes LD, McCue P, Robinson AK, Eryomin V, Sard L, Tagliabue E, Greco A, Fusetti L, Schwartz G, Pierotti MA, Croce CM, Huebner K (1997) Replacement of FHIT in cancer cells suppressed tumorigenicity. Proc Natl Acad Sci USA 94: 13771–13776 9391102 10.1073/pnas.94.25.13771 PMC28382

[bib25] Slaughter M, Skejkal W (1953) Field cancerisation in oral stratified epithelium: clinical implications of multicentric origin. Cancer (Phila) 6: 963–968 13094644 10.1002/1097-0142(195309)6:5<963::aid-cncr2820060515>3.0.co;2-q

[bib26] Sozzi G, Veronese ML, Negrini M, Baffa R, Cotticelli MG, Inoue H, Tornielli S, Pilotti S, De Gregorio L, Pastorino U, Pierotti MA, Ohta M, Huebner K, Croce CM (1996) The FHIT gene at 3p14.2 is abnormal in lung cancer. Cell 85: 17–26 8620533 10.1016/s0092-8674(00)81078-8

[bib27] Sozzi G, Sard L, De Gregonio L, Marchetti A, Musso K, Buttita F, Tornielli S, Pellegrini S, Veronese ML, Manenti G, Incarbone M, Chella A, Angeletti CA, Pastorino U, Huebner K, Bevilaque G, Pilotti S, Croce CM, Pierotti MA (1997) Association between cigarette smoking and FHIT gene alterations in lung cancer. Cancer Res 57: 2121–2123 9187107

[bib28] Sozzi G, Pastorino U, Moiraghi L, Tagliabue E, Pezzella F, Ghirelli C, Tornielli S, Sard L, Huebner K, Pierotti MA, Croce CM, Pilotti S (1998) Loss of FHIT function in lung cancer and preinvasive bronchial lesions. Cancer Res 58: 5032–5037 9823304

[bib29] Sozzi G, Oggionni M, Alasio L, Conte D, Tavecchio L, Pilotti S, Spinelli P, Calarco G (2002) Molecular changes track recurrence and progression of bronchial precancerous lesions. Lung Cancer 37: 267–270 12234694 10.1016/s0169-5002(02)00079-x

[bib30] Tanaka H, Shimada Y, Harada H, Shinoda M, Hatooka S, Imamure M, Ishizaki K (1998) Methylation of the 5' CpG island of the FHIT gene is closely associated with transcriptional inactivation in esophageal squamous cell carcinomas. Cancer Res 58: 3429–3434 9699676

[bib31] Testa JR, Siegfried JM, Liu Z, Hunt JD, Feder MM, Litwin S, Zhou JY, Taguchi T, Keller SM (1994) Cytogenetic analysis of 63 non-small cell lung carcinomas: recurrent chromosome alterations amid frequent and widespread genomic upheaval. Genes Chromosomes Cancer 11: 178–194 7530487 10.1002/gcc.2870110307

[bib32] Tomizawa Y, Nakajima T, Kohno T, Saito R, Yamaguchi N, Yokota J (1998) Clinicopathological significance of FHIT protein expression in stage I non-small cell lung carcinoma. Cancer Res 58: 5478–5830 9850082

[bib33] Tsuchiya E, Nakamura Y, Weng SY, Nakagawa K, Tsuchiya S, Sugano H, Kitagawa T (1992) Allelotype of non-small cell lung carcinomas – comparison between loss of heterozygosity in squamous cell carcinoma and adenocarcinoma. Cancer Res 52: 2478–2481 1314694

[bib34] Yanagisawa K, Kondo M, Osada H, Uchida K, Takagi K, Masuda A, Takahashi T (1996) Molecular analysis of the FHIT gene at 3p14.2 in lung cancer cell lines. Cancer Res 56: 5579–5582 8971157

[bib35] Yokota J, Sugimura T (1993) Multiple steps in carcinogenesis involving alterations of multiple tumor suppressor genes. FASEB J 7: 920–925 8344488 10.1096/fasebj.7.10.8344488

[bib36] Zanesi N, Fidanza V, Fong LY, Mancini R, Druck T, Valtieri M, Rudiger T, McCue PA, Croce CM, Huebner K (2001) The tumour spectrum in FHIT-deficient mice. Proc Natl Acad Sci USA 98: 10250–10255 11517343 10.1073/pnas.191345898 PMC56947

[bib37] Zienolddiny S, Ryberg D, Arab MO, Skaug V, Haugen A (2001) Loss of heterozygosity is related to p53 mutations and smoking in lung cancer. Br J Cancer 84: 226–231 11161381 10.1054/bjoc.2000.1528 PMC2363705

[bib38] Zöchbauer-Müller S, Fong KM, Maitra A, Lam S, Geradts J, Ashfaq R, Virmani AK, Milchgrub S, Gazdar AF, Minna JD (2001) 5′ CpG island methylation of the FHIT gene is correlated with loss of gene expression in lung and breast cancer. Cancer Res 61: 3581–3585 11325823

